# Transfer of manualized Short Term Psychodynamic Psychotherapy (STPP) for social phobia into clinical practice: study protocol for a cluster-randomised controlled trial

**DOI:** 10.1186/1745-6215-12-142

**Published:** 2011-06-08

**Authors:** Jörg Wiltink, Christian Ruckes, Antje Haselbacher, Marco Canterino, Falk Leichsenring, Peter Joraschky, Frank Leweke, Karin Pöhlmann, Manfred E Beutel

**Affiliations:** 1 Clinic of Psychosomatic Medicine and Psychotherapy, University Medical Center of the Johannes Gutenberg University Mainz, Germany; 2 Interdisciplinary Centre for Clinical Trials (IZKS), University Medical Center of the Johannes Gutenberg University Mainz, Germany; 3 Department of Psychosomatics and Psychotherapy, University of Giessen, Germany; 4 Department of Psychosomatic Medicine and Psychotherapy, Medical Faculty Carl Gustav Carus, Technical University of Dresden, Germany

## Abstract

**Background:**

Psychodynamic psychotherapy is frequently applied in the treatment of social phobia. Nevertheless, there has been a lack of studies on the transfer of manualized treatments to routine psychodynamic practice. Our study is the first one to examine the effects of additional training in a manualized Short Term Psychodynamic Psychotherapy (STPP) procedure on outcome in routine psychotherapy for social phobia. This study is an extension to a large multi-site RCT (N = 512) comparing the efficacy of STPP to Cognitive-Behavioral Therapy (CBT) of Social Phobia.

**Methods/Design:**

The manualized treatment is designed for a time limited approach with 25 individual sessions of STPP over 6 months. Private practitioners will be randomized to training in manualized STPP vs. treatment as usual without a specific training (control condition). We plan to enrol a total of 105 patients (84 completers). Assessments will be conducted before treatment starts, after 8 and 15 weeks, after 25 treatment sessions, at the end of treatment, 6 months and 12 months after termination of treatment. The primary outcome measure is the Liebowitz Social Anxiety Scale. Remission from social phobia is defined scoring with 30 or less points on this scale.

**Discussion:**

We will investigate how the treatment can be transferred from a controlled trial into the less structured setting of routine clinical care. This question represents Phase IV of psychotherapy research. It combines the benefits of randomized controlled and naturalistic research. The study is genuinely designed to promote faster and more widespread dissemination of effective interventions. It will answer the questions whether manualized STPP can be implemented into routine outpatient care, whether the new methods improve treatment courses and outcomes and whether treatment effects reached in routine psychotherapeutic treatments are comparable to those of the controlled, strictly manualized treatment of the main study.

**Trial Registration:**

German Clinical Trials Register (DRKS) DRKS00000570

## Background

Social phobia (SP, often also termed social anxiety disorder, SAD) is a disorder marked by an irrational intense fear of social or performance situations in which embarrassment may occur. According to the DSM-IV [1] diagnostic criteria "exposure to the feared social situation almost invariably provokes anxiety, which may take the form of a situationally bound or situationally predisposed panic attack" by concomitant discernment "that the fear is excessive or unreasonable". The symptoms must cause clinically significant distress or impairment in social, occupational, or other important areas of functioning.

The average 12 months prevalence rate of social phobia in the German population is 2% [2,3]. Women are more likely than males to develop a social phobia. Mean age of onset is between age 10 and 16.6 years [4]. Social phobia is a chronic disorder usually accompanied by comorbid depression, personality disorders, other anxiety disorders or substance abuse [4]. Keller (2003) indicates, that only one-third of patients with social phobia attain full remission within 8 years [5]. Because the disorder is often mistaken as shyness, social phobia is often not recognized and therefore undertreated [4,5].

The quality and effectiveness of psychodynamic psychotherapy in clinical practice, conducted by more than 50% of certified psychotherapists [6] in Germany, is unknown for patients with social phobia. As manualized treatments for specific disorders have rarely been used in psychodynamic training and practice, many psychodynamic practitioners are likely to be biased against structured short-term treatment approaches. The comparatively recent diagnosis of social phobia has found little consideration in psychodynamic research and practice. It is unknown whether new treatment approaches such as those evaluated in the first funding period of the Social Phobia Network (Sopho-Net funded by the German Federal Ministry of Education and Research, BMBF [7]) will improve the effects of routine psychodynamic psychotherapy. Therefore it is important to investigate how this new treatment method can be transferred from controlled trials into the less structured setting of routine clinical care, and whether the health care system benefits from such developments. This question represents phase IV of psychotherapy research.

## Methods/Design

### Study centres

The study is being carried out at three trial sites. The participating centres are the Clinic for Psychosomatic Medicine and Psychotherapy of the University Medial Centre, Johannes Gutenberg University, Mainz, the Department of Psychosomatics and Psychotherapy, University of Giessen and the Department of Psychosomatic Medicine and Psychotherapy, Medical Faculty Carl Gustav Carus, Technical University of Dresden.

In contrast to other RCTs in this trial patients are not recruited by centres. Participating therapist announce patients potentially fulfilling inclusion criteria and willing to participate to the trial site.

For recruitment of therapists all officially listed (chambers of psychotherapists and medical doctors) psychodynamic psychotherapists in the regions of the trial sites are asked for willingness to participate as study therapists. All therapists underwent a specific training (about 5 years of training) in psychodynamic psychotherapy. Due to the intended naturalistic character of our study we have defined relatively broad inclusion criteria for the participating therapists. Therapists can be of any age and gender. Double certification in CBT is not an exclusion criterion, as it is relatively rare in the German health care system. Therapists from the first funding period - specifically trained in STPP for Social Phobia are excluded.

Therapeutic experience (including former training in SET) and allegiance to the treatment condition will be assessed.

### Participants

Patients' inclusion and exclusion criteria are listed in table 1. According to these criteria, the sample of patients included in the study is representative for patients suffering from social phobia who qualify for treatment in an outpatient setting. No subject will be allowed to enrol in this trial more than once. Over a period of three years, a total of 105 patients are planned to be enrolled in the study (35 patients in each centre). A minimum number of 84 completers is intended.

**Table 1:** Inclusion and exclusion criteria

Inclusion criteria	Diagnosis of SP (SCID-I [8]) and Liebowitz Social Anxiety Scale >30 (>60 for generalized subtype) [9] age: 18 to 70 years SP must be primary diagnosis (most severe disorder according to ADIS-IV) SP patients with comorbid disorders will be included, provided that SP is the primary diagnosis, thus ensuring a clinically representative sample as well as analyses of subgroups (e.g. type of SP, patients with comorbid depressive disorder) Informed consent.
**Exclusion criteria**	psychotic disorder prominent risk of self-harm acute substance related disorders personality disorders except for cluster C: avoidant, obsessive-compulsive or dependent personality disorder (SCID-II) organic mental disorder severe medical conditions concurrent psychotherapeutic treatment psychopharmacological treatment (stable medical treatments; e.g. SSRI without dose adaptation are permitted)

### Interventions

The manualized treatment approach is based on Luborsky's Supportive Expressive Therapy (SET). The treatment includes the characteristic elements of SET therapy, that is, setting goals, focus on the Core Conflictual Relationship Theme (CCRT) associated with the patient's symptoms, interpretive interventions to enhance insight into the CCRT, and supportive interventions, in particular fostering a helping alliance. In order to tailor the treatment more specifically to social phobia, treatment elements have been added, for example informing the patient about the disorder and the treatment, a specific focus on shame and on unrealistic demands, and encouraging the patient to confront anxiety-provoking situations. More directive interventions are included as well, such as specific prescriptions to stop persisting self-devaluations [10]. The treatment is designed for a time limited approach with 25 individual sessions of STPP over 6 months (as reimbursed by German health insurance).

Those patients who are treated in the control condition will receive standard psychodynamic treatment.

### Assessment

**Figure 1:**
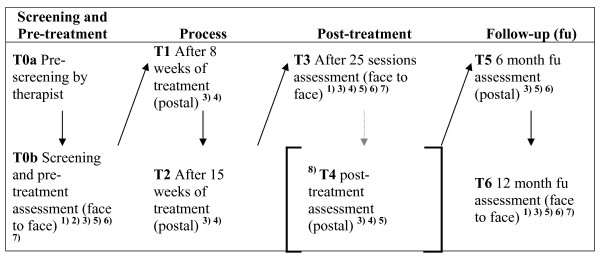
**Time points of assessment**. ^1) ^SKID-Interview, CGI, LSAS-Interview, ^2) ^Informed consent, check of inclusion criteria, ^3) ^LSAS questionnaire, ^4) ^process measures (e.g. HAQ, patient self-rating of therapist's adherence to treatment manual), ^5) ^BDI, ^6) ^EQ-5D, CSSRI, ^7) ^SPAI, FSKN, DKB-35, CDS, BSSS, ^8) ^if treatment exceeds 25 sessions additional post-treatment assessment is performed immediately after termination

The time points of assessment are shown in Figure 1. Assessments will be conducted before treatment starts, after 8 and 15 weeks, after 25 treatment sessions, at the end of treatment, 6 months and 12 months after termination of treatment. If treatment exceeds 25 sessions additional post-treatment assessment is performed immediately after termination.

Patients are assessed by independent and trained SCID interviewers, who are blind to the treatment condition.

### Objectives and hypotheses

The research questions of this trial are:

(1) How can manualized STPP be implemented into routine outpatient care?

(2) Will the new methods lead to an improvement of treatment courses and outcomes?

(3) Will treatment effects reached in routine psychotherapeutic treatments be comparable to those of the controlled, strictly manualized treatment of the main study?

The major hypotheses are: (1) Treatment effects reached by private practitioners trained with the manualized procedure of STPP of the main trial will be superior to therapists who apply their usual psychodynamic treatment. (2) Implementation of manualized STPP will lead to an average reduction of treatment duration and costs.

Further objectives: a) direct comparison (using the same measures) of results based on an efficacy and an effectiveness design; b) comparison between effectiveness in different fields of health care delivery. In combination with the results of the RCT from the first funding period, this study will allow to estimate the reduction of treatment duration and costs made possible through implementation of treatment manuals derived from the main trial into clinical practice.

Furthermore, this trial has been planned in close collaboration with the parallel trial for short-term cognitive-behavioural therapy (CBT). Endpoints and outcome measures were chosen in accordance with the main trial [7] to allow comparisons between outcomes of the RCT and the transfer studies included in this psychotherapy research network. Outcome measures include internationally used valid and highly relevant measures of SP.

### Outcomes

The primary outcome measure is the Liebowitz Social Anxiety Scale (LSAS [9]) ≤30 indicating remission from social phobia. The primary endpoint is the assessment after 25 sessions. Secondary outcome measures include another scale for the assessment of social anxiety (SPAI [11]) and scales for clinical global impression (CGI [12]), depression (BDI [13]), self-image (FSKN [14]), body image (DKB-35 [15]), depersonalization (CDS [16]), social support (BSSS [17]), quality of life and social functioning (EQ-5D [18]), Helping alliance (HAQ [19]), and therapists treatment adherence judged by patients and therapists. For this purpose self-rating scales for patients and therapists based on J. Barber's Penn Adherence Competence Scale (PACS-SE [20]) were constructed. The patient version has been applied in the first funding phase of Sopho-Net.

Furthermore, costs of treatment of Social Phobia are assessed with the German adaptation of the client sociodemographic and service receipt inventory (CSSRI [21]). This questionnaire includes information on health care utilization and loss of productivity. All instruments will be applied in both groups of this trial. Using established cut-off scores for LSAS, the percentages of patients defined as remitted will be assessed and statistically compared between the group treated by specifically trained therapists and the group receiving standard psychodynamic treatment.

### Sample size calculation

Being aware of the controversy whether RCT and efficacy studies reach the same effect sizes we used two recent meta-analyses on RCTs in STPP to estimate expected effect sizes. Abbass et al. (2006) [22] included trials on STPP with the majority of them (8 out of 13) not reporting manualized treatments. This condition comes closest to our comparison group receiving standard psychodynamic treatment. For general psychiatric symptoms effect sizes were medium (short term outcome d = .42, long term outcome d = .51). Based on the fact that effect sizes for specific target problems are usually larger, both treatment conditions will be expected to be superior to treatment as usual or no treatment. Both treatments seem to be highly acceptable and detrimental effects are not expected.

In their meta-analysis, Leichsenring et al. (2004) [23] reported effect sizes (prae-post) of d = .90 for the reduction of general psychiatric symptoms by STPP based on strictly manualized treatments. Compared to the results of Abbass et al. (2006) [22] with mostly non-manualized STPP we expect the between group effect size to be medium (about d = .48). Therefore, we pragmatically expected an effect size of 0.50 between the two groups (trained versus non trained therapist). In order to detect this difference at alpha = .05 (one-tailed) with a power of 0.80, N = 42 (one-tailed) patients per group are required according to the formula of Campbell et al. (2001) [24] for Cluster-Randomization, given that an average of 2 patients per therapist will be treated:

(z_α _= z-value for probability of error type I alpha, z_β _= z-value for probability of error type II beta, σ = standard deviation of outcome, E = expected ES of outcome, m = mean observations for each cluster, ρ = Intracluster correlation coefficient (ICC), ES = E/σ = standardized effect size)

While planning this study no meaningful published data on remission rates for manualized vs. non-manualized psychodynamic psychotherapy of social anxiety disorders were available. We based sample size calculation on available meta-analyses on psychodynamic psychotherapy all based on continuous outcomes. Estimating the sample size needed for a dichotomous approach (remission rates), we introduced the following conservative assumptions:

a) The ICC was set to 15%, which is high for comparable studies. According to Campbell (2001) [24] the ICC for outcome variables could be assumed to be less than 5%.

b) The average number of patients in a cluster was assumed to be 2. In fact, due to the prevalence of the disease we expect the average number of patients in a cluster to be less than 2.

c) Within the planning the sample size of 42 per group was calculated for patients completing the trial. For the primary analysis (ITT) drop-outs will be analysed as patients without remission. When assuming less than 50% remitters and an equal drop-out rate in both treatment groups, the absolute difference between remission rates will not change, but the relative difference will be much greater and will enlarge the difference between treatments substantially.

At a conservative drop-out rate of 25% (taking into account slightly elevated drop-outs in a practice study), a total of N = 105 patients are required to be allocated to the trial. Thus, N = 35 patients will have to be included in each centre. According to the data of the Davidson et al. (2004) [25] study with social phobia patients, N = 257 patients would have to be assessed for eligibility. As all patients have already received a clinical diagnosis by the practitioner before undergoing the standardized interview, we estimate that not more than about 250 patients will have to be assessed for eligibility.

### Randomization

Therapists will be randomly assigned either to a training group in which they will undergo an intensive training of the short-term STPP manual for social phobia as developed for the multi-centre study or to a control group in which the non-manualized standard psychodynamic treatment is applied. Randomization will be stratified taking into account the three trial sites. Randomization of therapists will be performed centrally by IZKS Mainz before training of the therapists starts. Patients will not be randomized and will not know whether their therapist was recently trained in STPP for SP or not.

### Statistical analysis

#### Primary analysis

The following hypothesis will be tested:

H_0_: π_manualized _≤ π_standard _vs. H_1_: π_manualized _> π_standard_

where π_manualized _and π_standard usual _are the true remission probabilities in the manualized treatment group and the standard routine care group after treatment period respectively.

Remission rates will be compared between specifically trained STPP (manualized) and psychodynamic treatment as usual (standard) group by logistic regression with covariates for treatment, sex of the therapist, and experience of the therapist. Due to the cluster randomized nature of the study a term for the therapist will be added. Within each therapist compound symmetry will be assumed. Intent-to-treat analyses (LOCF) as well as completer analyses will be conducted. Drop-outs will be considered as non-responders/non-remitters in the analysis. As sensitivity analyses a chi-square test and a conditional logistic regression adjusted for the therapist will be performed. Also the LSAS will be analysed as a continuous measure by means of mixed model with repeated measurements for the ITT population. Replacement strategies of missing values will be discussed after assessing the pattern of the missing value structure.

#### Secondary analyses

Self-report questionnaires and observer ratings (SPAI, CGI, BDI, FSKN, DKB-35, CDS, BSSS, EQ-5D, HAQ, treatment adherence) will be analysed by mixed models with repeated measurements. Further analyses will include

• cross-sectional analyses comparing therapists;

• subgroup analyses of subjects' and therapists' conditions

• costs of the SP treatment (CSSRI)

All analyses will be conducted on a one-sided level of significance of 0.05. Descriptive statistics showing the measurements over time will be presented whenever appropriate.

Serious adverse events and drop-outs will be analysed descriptively.

### Safety aspects

Safety parameters will comprise newly occurring psychiatric diagnoses (SCID-I) and all serious adverse events that are reported during and up to six months after treatment.

### Medical Complications

The recording of adverse events will be restricted to psychological conditions. Formally, they are defined as any disorder classified by the International Classification of Diseases F00-F99 ("Mental and Behavioral Disorders").

A serious adverse event (SAE) is an adverse event that may occur at any time of the treatment phase or up to 6 months after end of treatment: results in death; is life-threatening; requires subject hospitalization or prolongation of existing hospitalization; results in persistent or significant disability/incapacity; is a congenital anomaly/birth defect

Changes in mental disorders according to ICD-10 F00-F99 will be specifically asked for in the CRF pages filled by the interviewer at the following time-points: after 25 session of treatment; twelve months after end of treatment.

Any AE (according to the study specific definition) reported by the subject or detected by the local investigator will be collected during the trial and must be documented in the CRF. ICD-10 will be used by the local investigator to code the event. The standard coding system for adverse events, MedDRA, has been reported to be inadequate in psychotherapeutic studies [26]. The clinical course of the AE will be followed until it has changed to a stable condition or until end of follow-up phase, whatever comes first.

In case of SAEs Ethics Committee (EC) and Data Safety Monitoring Board (DMSB) will be informed within 24 hours after the SAE becomes known.

### Ethical issues

The final study protocol and the final version of the written informed consent form were approved by the Ethics Committee of the Federal State of Rhineland Palatine (Germany), which is responsible for the Principal Investigator (Ref. No. 037.249.10 [7258]).

The procedure set out in this protocol, pertaining to the conduct, evaluation, and documentation of this trial, were designed to ensure that all persons involved in the trial abide by Good Clinical Practice (GCP) and the ethical principles described in the current revision of the Declaration of Helsinki. The trial will be carried out in keeping with local legal and regulatory requirements.

Before being admitted to the clinical trial, patients must consent to participate after the nature, scope, and possible consequences of the clinical trial have been explained in a form understandable to them. The patients must give written informed consent to participate in the study including their consent to publish.

## Discussion

The study is designed as an extension to the larger multi-site project in which Cognitive-Behavioral Therapy (CBT) and Short Term Psychodynamic Psychotherapy (STPP) of Social Phobia based on SET have been evaluated [7]. It combines the benefits of randomized controlled trials and naturalistic studies. We will ascertain to what degree practitioners integrate the new methods into their clinical routine. Furthermore, the study will determine whether the new methods lead to an improvement of treatment course and outcome and also whether the treatment effects of manualized STPP in routine psychotherapeutic treatment are comparable to those of the controlled, strictly manualized treatments of the main study. This study estimates potential reductions of treatment duration and costs by implementation of treatment manuals into clinical practice.

In contrast to the previous RCT in this study the patients are not recruited by the participating centres. Patients will seek treatment in the regular way of the German health care system attending private practitioners. Therefore, participating therapists recruit patients and announce them to the study centres. In the previous RCT treatments were limited to 25 sessions. In our study therapists are completely free to determine treatment duration. Accordingly, we determined primary outcome after 25 sessions. Our comparison group is naturalistically treated with standard psychodynamic psychotherapy without any additional training of the therapists.

Examinations of these issues in a methodologically rigorous manner will be of high relevance not only for the domain of social phobia but for the dissemination of innovative psychotherapeutic treatments in general.

Implementing the best obtainable treatment into the practice of experienced psychotherapists can be expected to increase treatment effectiveness. While evidence for the efficacy of Supportive Expressive Psychotherapy (SET) and other variants of Short Term Psychodynamic Psychotherapy (STPP) has accumulated [22,23], there has been a lack of studies on the transfer of manualized treatments to routine psychodynamic practice. Our study will be the first one to directly examine the effects of an additional training in a manualized and highly effective STPP procedure on outcome in routine psychotherapy for social phobia.

## List of abbreviations

ADIS-IV: Diagnostic interview; AE: Adverse event; BDI: Beck Depression Inventory; BMBF: Federal Ministry of Education and Research; BSSS: Berlin Social Support Scale; CBT: Cognitive Behavioral Therapy; CCRT: Core Conflictual Relationship Theme; CSSRI: Client Sociodemographic and Service Receipt Inventory; CDS: Cambridge Depersonalization Scale; DKB: Dresdner Körperbildfragebogen; CRF: Case Report Form; DMSB: Data Monitoring and Safety Board; DSM: Diagnostic and Statistical Manual of Mental Disorders; EC: Ethic Committee; EQ-5D: Quality of life questionnaire; ES: Effect size; FSKN: Frankfurt self concept scales; HAQ: Helping Alliance Questionnaire; ICC: Intracluster Correlation Coefficient; ICD-10: International Classification of Diseases; ITT: Intention to treat; IZKS: Interdisciplinary Centre for Clinical Trials; LOCF: Last observation carried forward; LSAS: Liebowitz Social Anxiety Scale; MedDRA: Medical Dictionnary for Regulatory Activities; RCT: Randomised Controlled Trial; SAD: Social Anxiety Disorder; SAE: Serious Adverse Event; SCID: Structured Clinical Interview for DSM Disorders; SET: Supportive Expressive Therapy; SP: Social Phobia; SPAI: Social Phobia and Anxiety Inventory; SSRI: Selective serotonin reuptake inhibitors; STPP: Short Term Psychodynamic Psychotherapy

## Competing interests

The authors declare that they have no competing interests.

## Authors' contributions

JW did the first draft of the manuscript. JW, FL and MEB did the final draft of the manuscript and critically revised it for its intellectual content. JW, CR, AH, MC, FL, PJ, KP and MEB substantially contributed to the conception and the design of the study. All authors read and approved the final manuscript.
